# Association Between Perceived Control and Acute Coronary Syndrome Symptom Severity: A Prospective Cohort Study

**DOI:** 10.1002/nop2.70181

**Published:** 2025-03-14

**Authors:** Fatma Refaat Ahmed, Fiona Timmins, Rawia Gamil, Nabeel Al‐Yateem, Mary Ryder, Heba Mustafa, Mohannad Eid AbuRuz

**Affiliations:** ^1^ Department of Nursing College of Health Sciences, University of Sharjah Sharjah UAE; ^2^ Department of Critical Care and Emergency Nursing Faculty of Nursing, Alexandria University Alexandria Egypt; ^3^ School of Nursing, Midwifery & Health Systems University College Dublin Dublin Ireland; ^4^ Hind Bint Maktoum College of Nursing and Midwifery Mohammed Bin Rashid University of Medicine and Health Sciences, Dubai Health Dubai UAE

**Keywords:** acute coronary syndrome, analysis of variance, chest pain, control groups, dyspnoea, Egypt, fatigue, perceived control, prospective studies, symptoms

## Abstract

**Aim:**

To determine whether there are differences in patterns of symptom severity in three major ACS symptoms (i.e., chest pain, fatigue, and dyspnoea) over the days of CCU stay between patients with higher and lower levels of perceived control.

**Design:**

A prospective cohort study with 3 days of follow‐up.

**Methods:**

Hundred and thirty‐five patients were followed prospectively for 3 days, collecting data on (1) perceived control, using the Arabic version of the Controlled Attitude Scale (CAS‐R); and (2) symptom severity, using a symptoms diary. A mixed repeated measure design ANOVA was used for data analysis.

**Results:**

Comparative analysis of the high‐ and low‐perceived control groups revealed that there was a significant reduction in chest pain, fatigue, and dyspnoea symptom severity over the course of the 3 days for both groups. Compared to the low‐perceived control group, the high‐perceived control group had significantly lower chest pain on day 3, fatigue on days 2 and 3, and dyspnoea on all days.

**Conclusion:**

Patients with high levels of perceived control have lower levels of symptom severity than those with low perceived control. Interventions to improve perceived control among this population are highly recommended.

**Patient or Public Contribution:**

No patient or public contribution.

## 1 Introduction

Deaths and disability‐adjusted life years (DALYs) due to coronary artery disease (CADs) are the leading causes of disability and mortality globally, and a narrative review reported that the increasing load of longitudinal disability rates associated with cardiovascular diseases (CVDs) falls mainly on low‐ and middle‐income countries, accounting for nearly 7 million deaths and 129 million DALYs annually (Ralapanawa and Sivakanesan 2021). From 2011 to 2020, the mortality rate related to CADs increased by 146% and 174% for women and men (respectively) in the Eastern Mediterranean Region (EMR) (Reda et al. 2020), where the rapid escalation of the CVDs pattern has been worrisome for some years (Turk‐Adawi et al. 2018). Egypt is the most populous country in the EMR region, with nearly 105 million people, and a 51% prevalence rate of premature Acute Coronary Syndrome (ACS) (i.e., with initial clinical manifestations at the ages of < 60 and < 55 years for females and males, respectively) (Reda et al. 2020). Moreover, the WHO CVD Risk Chart Working Group (2019) analysed data from 79 low‐ and middle‐income countries and found that the CVD risk among individuals aged 40–64 years in Egypt is estimated to be more than 16%.

Keeping in mind the high prevalence of CVDs in general, accurate symptom recognition and assessment can be important in assisting ACS patients to seek care in a timely manner, thereby preventing undesired complications, maximising their health outcomes, and reducing treatment costs.

## 2 Background

Patients usually perceive any form of cardiac event, including ACS, as intrinsically life‐threatening, precipitating an intense fear of death (Mentrup et al. 2020), and fatalistic responses such as a sense of hopelessness (Ivanovs et al. 2018), depression (Bahall 2019), anxiety (Ivanovs et al. 2018), and high levels of stress (Albert et al. 2017), all of which are manifest in relation to a feeling of helplessness and loss of control, which can be conceptualised as low patient self‐efficacy (von Känel et al. 2021). Various recent studies have reaffirmed that illness and symptom perception affect patients' health behaviours and subsequent outcomes and prognosis, including treatment adherence, and functional outcomes affecting the ACS recovery rate (Grauman et al. 2022; Karl et al. 2021; Sawyer et al. 2019). In this regard, the ‘Common‐Sense Model of Self‐Regulation’ describes that individuals manage health threats (e.g., ACS) through a dynamic process, including representations of threats (i.e., symptoms recognition), and actions for addressing threats of outcomes to modify further efforts (Leventhal et al. 2016).

Perceived control is a strategy that could help in improve patient's recovery post‐ACS (AbuRuz 2018). Perceived control is understood in terms of people's beliefs in their ability to proactively deal with adverse events (Moser and Dracup 1995; Thompson et al. 1993). Perceived control significantly affects cardiac patients' mental health, and empirical studies attest that it can improve quality of life (QoL) among cardiac patients (Kondo, Oki, et al. 2021; AbuRuz and Al‐Dweik 2022). Perceived control also moderates the relationship between anxiety and in‐hospital complications after ST segment elevation myocardial infarction (AbuRuz 2018). In general, high levels of perceived control are associated with optimum physical and emotional outcomes in patients with CVDs (AbuRuz 2019; Castillo‐Mayén et al. 2021).

In the first day of Coronary Care Unit (CCU) admission, it might be difficult to check the effect of perceived control on the symptom severity due to the nature of the disease (i.e., ACS). For this reason, many previous studies in this field checked its effect using a cross‐sectional design (particularly measuring very early in the disease process), and did not find any significant associations (Achmad et al. 2023; Allan et al. 2007; Kondo, Eckhardt, et al. 2021; Zou et al. 2023). We attained similar results in the current study on the first day, since we did not find any difference between the studied groups (as described in detail below). However, when the patients became more engaged and started to apply their perceived control in addition to the regular nursing management (i.e., on the second and third days), we attained significant results. For this reason, we used the prospective design, which constitutes a major difference between this study and previous research in this field.

However, the impact of perceived control on symptom status has not been well studied using a prospective approach; thus, this prospective cohort study seeks to determine whether there are differences in patterns of symptom severity in three major ACS symptoms (i.e., chest pain, fatigue, and dyspnoea) over 3 days of CCU stay between patients with higher and lower levels of perceived control.

### 2.1 Research Question

Is there a difference in symptom severity (chest pain, fatigue, and dyspnoea) based on perceived control (low and high) levels during the first 3 days of the disease course?

## 3 Methods

### 3.1 Research Design

A prospective cohort study with 3 days of follow‐up.

### 3.2 Study Settings

Patients were recruited from CCUs of three university hospitals of Alexandria University, Egypt, and they were followed by the researchers until discharge. The hospitals are run by the Ministry of Higher Education and Scientific Research and serve as teaching facilities, providing services to the general public and being connected with government universities. The bed occupation rate is always high, as they provide free services to patients of any age and gender from all over the country who are not covered by national health insurance.

### 3.3 Study Participants

**FIGURE 1:**
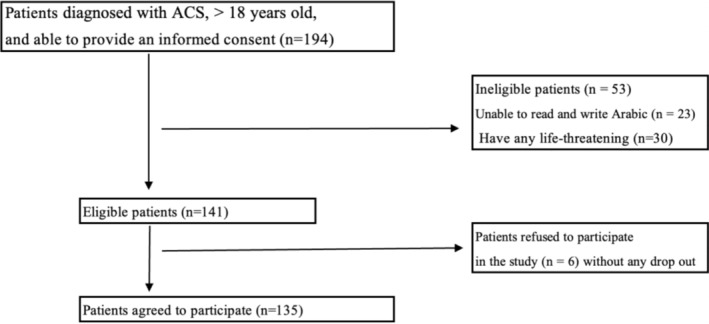
ACS sampling flowchart.

Patients were enrolled in this study subject to the following inclusion criteria: (1) having a cardiologist‐confirmed diagnosis of ACS (ST‐Elevated Myocardial Infarction (STEMI), Non‐ST Elevated Myocardial Infarction (NSTEMI), and unstable angina), (2) being aged 18 and above, (3) being able to read and write in Arabic, and (4) not having any life‐threatening dysthymias or obvious cognitive impairments (Figure 1). Patients who had any life‐threatening dysthymias (e.g., ventricular fibrillation or ECG results indicative of myocardial myopathies) were excluded from this study. Also, eligible patients who developed hemodynamic instability for any reason within the three‐day period of the study were excluded. The sample size was calculated by G* power software (3.1) using the following criteria: type 1 error of 0.05, a power of 0.8, medium effect size (0.5), and using the following statistical test (F test): mixed repeated measure design analysis of variance (ANOVA) for RQ; based on that, the number of needed participants was 128, so we recruited 135 participants to account for dropout. It is worthy to note that all participants completed the survey without any dropout.

### 3.4 Instruments

#### 3.4.1 Demographic and Clinical Variables

The following demographic characteristics were collected either during patients' interview sessions or by medical records review to control for potential confounding variables (Kondo, Oki, et al. 2021; AbuRuz and Al‐Dweik 2022; AbuRuz 2019): age, sex, residence site, marital status, occupation, monthly income, education level, presence of social support (i.e., involvement of one of family members and/or significant person with the patient in their care plan), body mass index (BMI), family history of coronary heart disease (CHD), history of smoking, medication regimen, and history of comorbidities (diabetes mellitus, hypertension, heart failure, previous acute myocardial infarction, and hyperlipidaemia).

#### 3.4.2 Perceived Control

The Arabic version of Controlled Attitude Scale (CAS‐R) was adopted from AbuRuz (2019). It includes eight items answerable with a five‐point Likert scale, from strongly agree (5) to strongly disagree (1). This scale is used to measure patients' perceived control which is refers to the individual own beliefs toward their ability to cope with stressful and emotional events (e.g., ACS), for example, ‘I have considerable ability to control my symptoms’ and ‘I can do a lot of things myself to cope with my heart condition’. Using translation and back‐ translation, AbuRuz (2019) checked the appropriateness of the items to Arab culture, and confirmed that the tool is easily understood by Arabic‐speaking participants. Upon producing the final version, construct validity was supported by hypothesis testing and factor analysis. According to AbuRuz (2019), the Cronbach's alpha of the scale is 0.85. According to previous studies, there are no published mean norms for this tool, so researchers conventionally use the median of their samples as a cut‐off point to classify patients as having high and low perceived control (Moser and Dracup 1995; McKinley et al. 2012).

#### 3.4.3 Symptom Severity

A symptoms' diary was used to allow patients to self‐report the severity of three symptoms (chest pain, fatigue, and dyspnoea) during their hospitalisation via face‐to‐face interviews. Symptom severity was rated using a 10‐point numerical analogue scale, with 0 indicating the absence of symptoms and 10 indicating the worst level of symptoms.

### 3.5 Ethical Considerations

The study was approved by (REDACTED) Research Ethics Committee (approval number: 2022‐9‐34) and the selected hospitals prior to data collection. Written informed consent was obtained and signed by each participant before the data collection, after a full explanation of the study purpose and participant rights, including the right to withdraw without their healthcare services or statutory rights being affected. The consent process included permission to review participants' medical records. Confidentiality was maintained through the study by applying an anonymous coding system. Moreover, all data were kept in private password‐protected computer files at the main researcher's office at the University of Sharjah. Aggregate data were used for publication purposes without any identifiers. Permission was obtained from the creator of the Arabic version of the Controlled Attitude Scale (CAS‐R) to use the instrument.

### 3.6 Procedure

The second author (holding a Ph.D. degree in critical care) met with the head nurses of the CCUs in the selected hospitals and explained the study's nature and purpose; then they met with all patients who met the inclusion criteria (as identified by the head nurses) to explain the study and invite them to participate. Patients who met the inclusion criteria and who agreed to participate signed an informed consent. When patients were haemodynamically stable, they were asked to complete socio‐demographic questionnaires and CAS‐R. After that, the second author provided patients with a symptoms diary to rate their symptoms from Day 1 to Day 3 post‐ACS event, and the patients were monitored frequently to ensure they completed the diary. Patients were asked to rate symptoms at the same time every day. Data were collected from April 2022 to September 2022.

### 3.7 Data Analysis

Data analysis was undertaken using SPSS software (v. 26.0) (IBM, Armonk, NY, USA). Descriptive statistics were analysed using means, standard deviations, numbers, and frequency distributions; the research question was definitively answered by a mixed repeated measure design ANOVA; perceived control was divided into high and low levels based on the median score of 28, as applied in previous studies (Moser and Dracup 1995; AbuRuz 2019; McKinley et al. 2012), whereby perceived control scores of > 28 and ≤ 28 were considered high or low (respectively).

## 4 Results

This section describes participants' characteristics, including levels of perceived control, and tests the results to answer the research question.

### 4.1 Participant Characteristics

A total of 135 patients participated in the study, of whom 81 had a high perceived control level, and 54 had a low perceived control level. The overall mean age was 52.43 ± 6.81 years, with nearly three‐quarters of the sample being males. The majority of the sample was working, had a history of hypertension and hyperlipidaemia. Table 1 shows that the only significant difference between high and low perceived control groups is the level of perceived control. All other characteristics did not differ between the two groups.

**TABLE 1:** Sociodemographic and clinical characteristics of the sample based on perceived control levels.

	Total sample (*N* = 135)	High perceived control (*n* = 54)	Low perceived control (*n* = 81)	*p* for T or *X* ^2^
Age	52.43 ± 6.81	51.87 ± 7.78	52.81 ± 6.11	*t* = 0.78, *p* = 0.44
Perceived control score	27.56 ± 2.39	29.92 ± 1.01	25.99 ± 1.61	< 0.001*
BMI	27.46 ± 7.51	27.78 ± 11.40	27.24 ± 2.86	*t* = −0.4, *p* = 0.69
Sex				*X* ^2^ = 0.1, *p* = 0.46
Male	98 (72.6)	40 (74.1)	58 (71.6)	
Female	37 (27.4)	14 (25.9)	23 (28.4)	
Marital status				*X* ^2^ = 0.53, *p* = 0.3
Married	85 (63.0)	32 (59.3)	53 (65.4)	
Single/divorced/widowed	50 (37.0)	22 (40.7)	28 (34.6)	
Working status				*X* ^2^ = 2.2, *p* = 0.11
Working	115 (85.2)	43 (79.6)	72 (88.9)	
Retired/not working	20 (14.8)	11 (20.4)	9 (11.1)	
Diagnosis				*X* ^2^ = 1.3, *p* = 0.19
Unstable angina	37 (27.4)	15 (27.8)	22 (27.2)	
NSTEMI	40 (29.6)	13 (24.1)	27 (33.3)	
STEMI	58 (43.0)	26 (48.0)	32 (39.5)	
History of DM	81 (60.0)	36 (66.7)	45 (55.6)	*X* ^2^ = 1.7, *p* = 0.2
History of HTN	117 (86.7)	49 (90.7)	68 (84.0)	*X* ^2^ = 1.3, *p* = 0.19
History of HF	35 (25.9)	18 (33.3)	17 (21.0)	*X* ^2^ = 2.57, *p* = 0.08
History of hyperlipidaemia	114 (84.4)	48 (88.9)	66 (81.5)	*X* ^2^ = 1.35, *p* = 0.18
History of pervious AMI	29 (21.5)	9 (16.7)	20 (24.7)	*X* ^2^ = 1.24, *p* = 0.19
Current smoker	72 (53.3)	29 (53.7)	43 (53.1)	*X* ^2^ = 0.05, *p* = 0.5
Family history of CHD	80 (59.3)	36 (66.7)	44 (54.3)	*X* ^2^ = 2.04, *p* = 0.11

*Note:* Values are M ± SD or *n* (%).

Abbreviations: AMI, acute myocardial infarction; BMI, body mass index; CHD, coronary heart disease; DM, diabetes mellitus; HF, heart failure; HTN, hypertension; NS, not significant; NSTMI, non‐ST elevated myocardial infarction; STMI, ST elevated myocardial infarction.

* To differntiate between the degree of significance levels.

### 4.2 Testing Research Question

**FIGURE 2:**
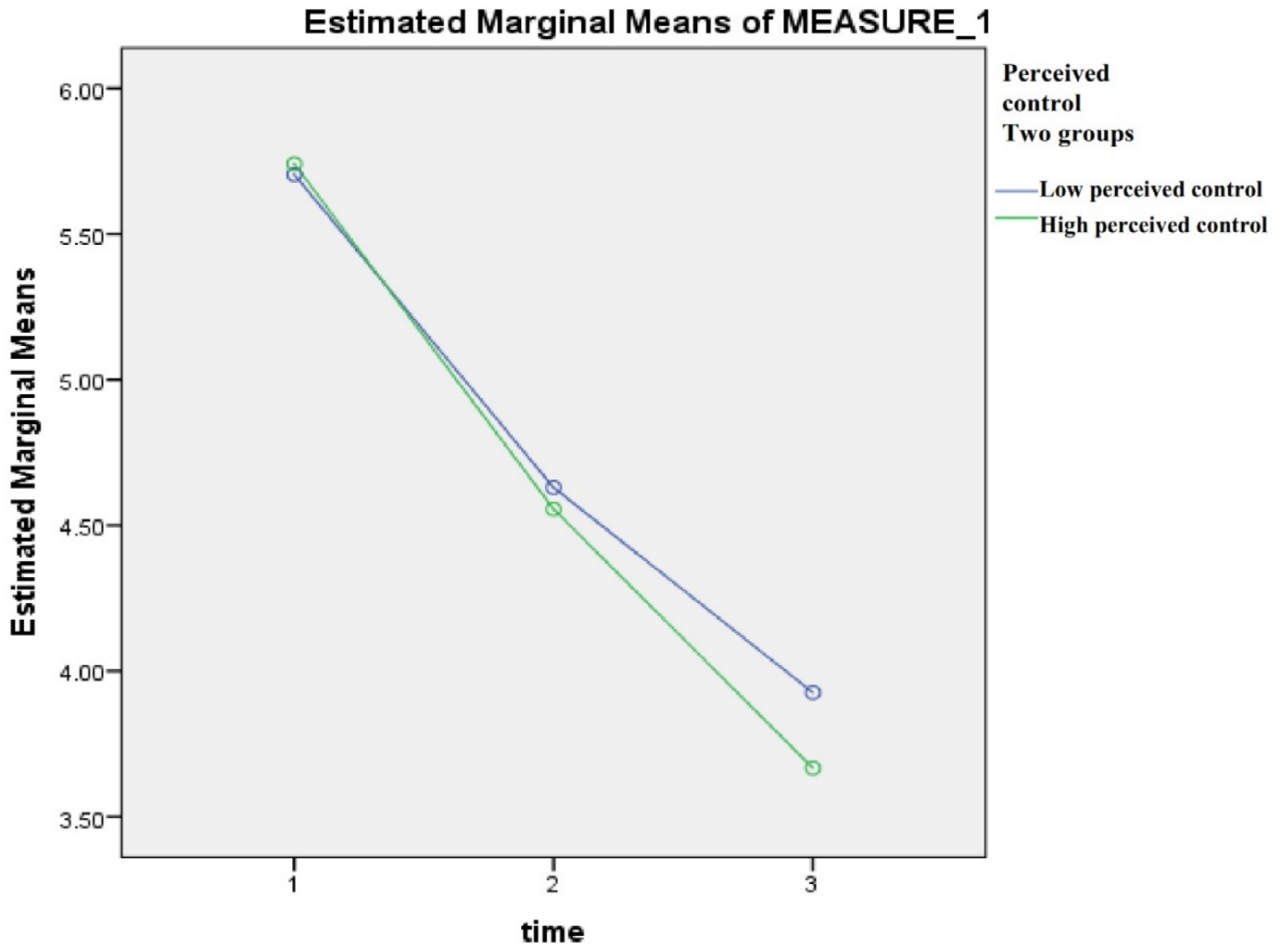
Estimated marginal means of chest pain, days 1–3.

**FIGURE 3:**
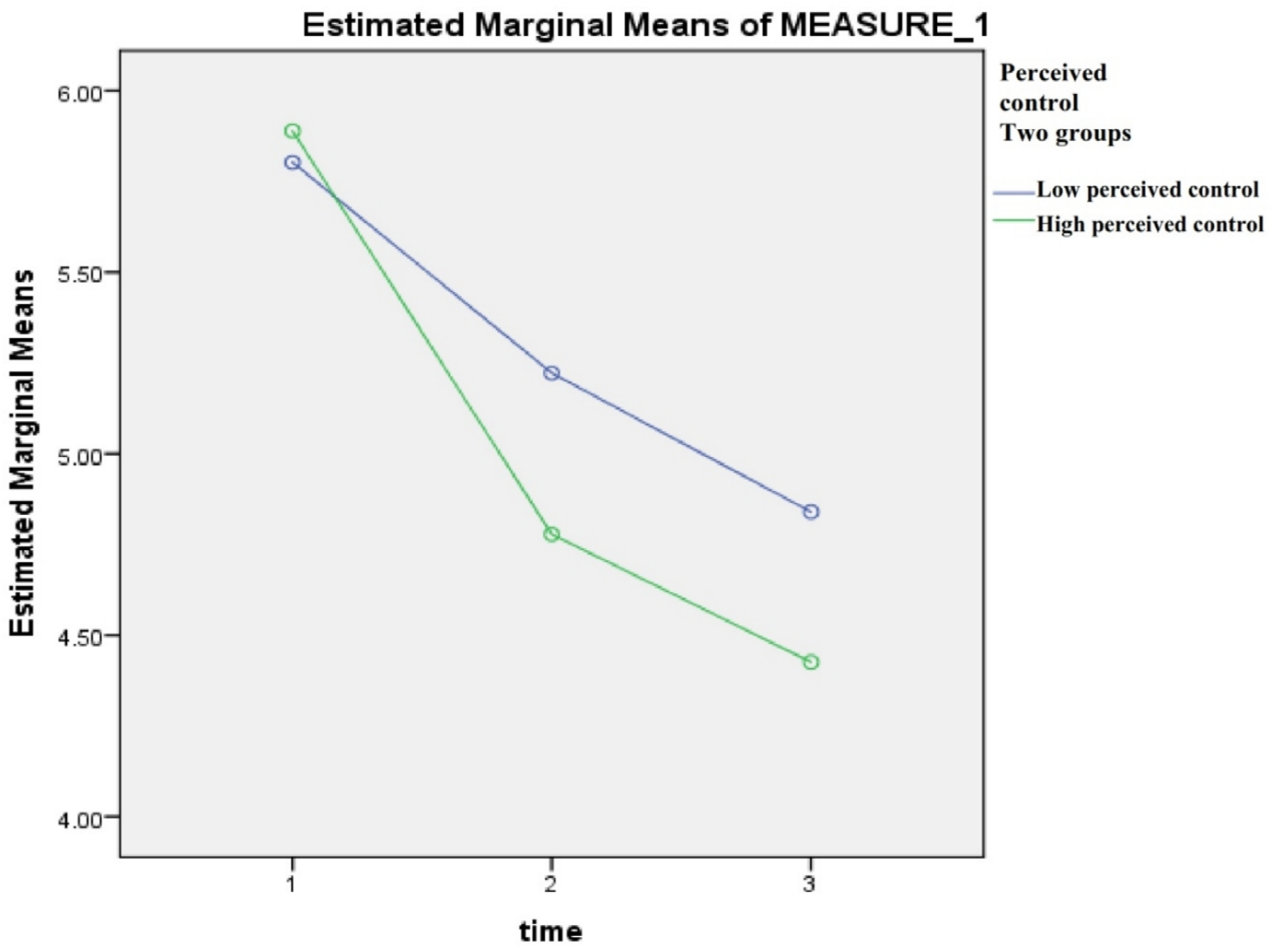
Estimated marginal means of fatigue, days 1–3.

**FIGURE 4:**
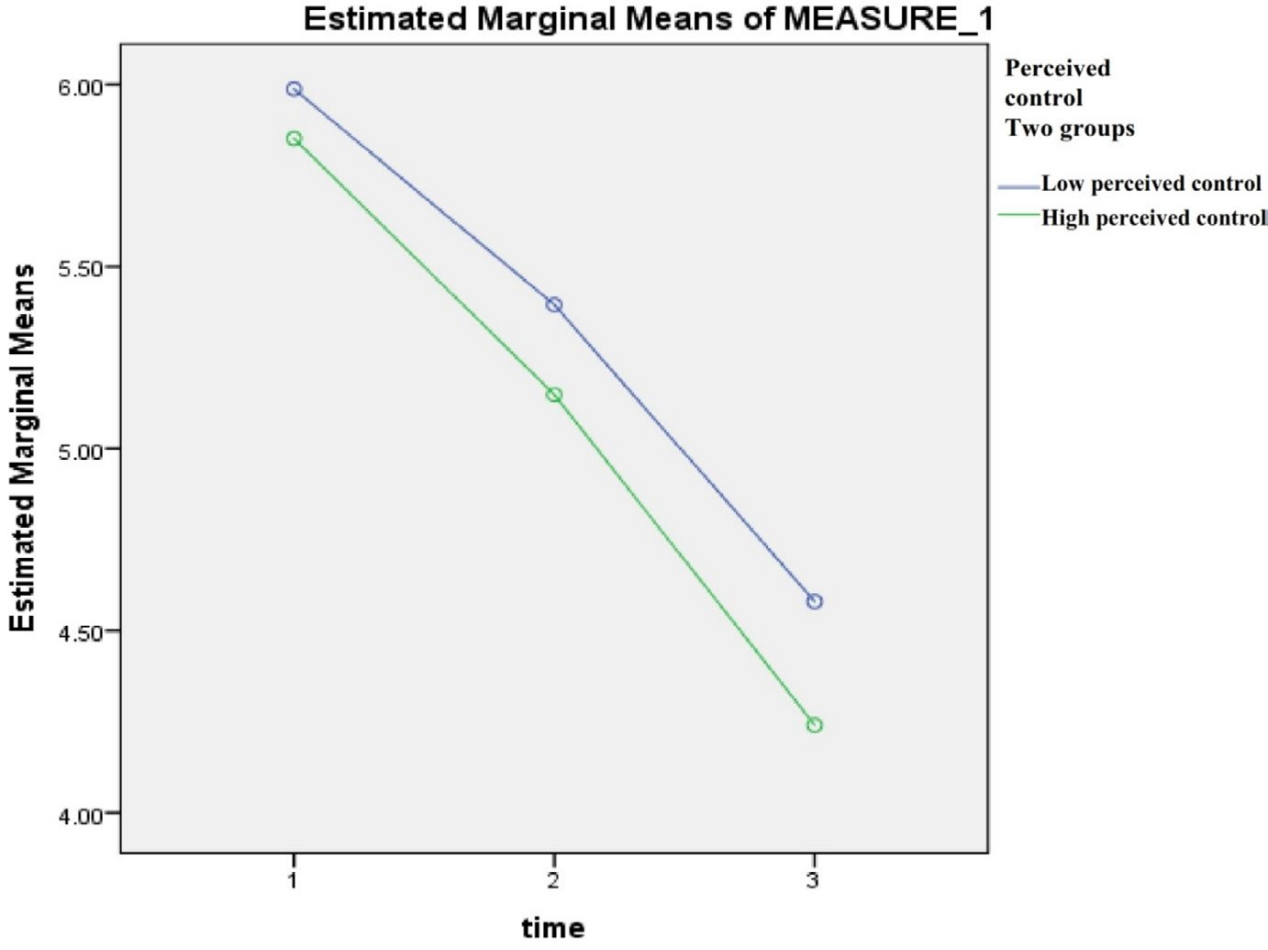
Estimated marginal means of dyspnoea, days 1–3.

Mixed‐model ANOVA testing was used to calculate the estimated marginal means (i.e., the difference in the means) of the dependent variables (chest pain, fatigue, and dyspnoea) in the high and low perceived control groups (independent variable) across Days 1–3 post‐ACS event. Table 2 shows that for both high and low perceived control groups there was a significant reduction in symptom severity (chest pain, fatigue, and dyspnoea) over the course of the 3 days, *p* < 0.001. However, when we look at the difference between the groups, we find that there were no significant differences between the high and low groups at days 1 and 2 in regard to chest pain level. Moreover, there was no statistically significant difference between the two groups at day 1 regarding fatigue levels. Other comparisons showed that patients who had high levels of perceived control had significantly lower chest pain on day 3, *p* = 0.04. Additionally, patients who had high levels of perceived control had significantly lower fatigue on days 2 and 3, *p* < 0.001. Dyspnoea was lower in the high perceived control group on all days (*p* = 0.003, 0.04, and 0.03, respectively) (Table 2, Figures 2, 3, 4).

**TABLE 2:** Comparison of chest pain, fatigue, and dyspnoea mean scores over time and between the high and low perceived control groups of the study using a mixed model ANOVA test (*N* = 135).

	Day 1 M1 (SD)	Day 2 M2 (SD)	Day 3 M3 (SD)	Sig. (between measurement)
Chest pain score				
High perceived control (*n* = 54)	7.74 (0.44)	4.56 (0.57)	3.67 (0.70)	M1 vs. M2, < 0.001** M1 vs. M3, < 0.001** M2 vs. M3, < 0.001**
Low perceived control (*n* = 81)	5.70 (0.46)	4.63 (0.53)	3.93 (0.54)	M1 vs. M2, < 0.001** M1 vs. M3, < 0.001** M2 vs. M3, < 0.001**
Sig. (between groups)	NS	NS	*p* = 0.04*	
Fatigue score				
High perceived control (*n* = 54)	5.89 (0.32)	4.78 (0.50)	4.42 (0.54)	M1 vs. M2, < 0.001** M1 vs. M3, < 0.001** M2 vs. M3, < 0.001**
Low perceived control (*n* = 81)	5.80 (0.40)	5.22 (0.47)	4.84 (0.46)	M1 vs. M2, < 0.001** M1 vs. M3, < 0.001** M2 vs. M3, < 0.001**
Sig. (between groups)	NS	< 0.001**	< 0.001**	
Dyspnoea score				
High perceived control (*n* = 54)	5.85 (0.41)	5.15 (0.57)	4.24 (0.78)	M1 vs. M2, < 0.001** M1 vs. M3, < 0.001** M2 vs. M3, < 0.001**
Low perceived control (*n* = 81)	5.99 (0.11)	5.40 (0.53)	4.58 (0.54)	M1 vs. M2, < 0.001** M1 vs. M3, < 0.001** M2 vs. M3, < 0.001**
Sig. (between groups)	*p* = 0.03*	*p* = 0.04*	*p* = 0.03*	

Abbreviations: M, mean; NS, not significant; SD, standard deviation.

* Significant at *p* value < 0.05.

** Significant at *p* value < 0.01.

**RQ:** Is there a difference in symptom severity (chest pain, fatigue, and dyspnoea) based on perceived control (low and high) levels during the first 3 days of the disease course?

## 5 Discussion

Most existing research on the relationship between perceived control and other health‐related variables was conducted in Western care settings, and more studies of diverse contexts are needed to assess the consistency of its associations across populations with different inherent socio‐cultural, economic, and healthcare system factors, all of which are likely to influence physiological and psychosocial well‐being. Egypt seems a particularly interesting setting, in that it is demographically significant and broadly representative of other Arab and EMR populations. Therefore, this study was conducted to add evidence to similar studies of the region (AbuRuz et al. 2019, 2022).

The main findings of this study indicate that homogeneous patients exhibited heterogeneous levels of perceived control. Thus, they were categorised into two groups based on the perceived control level: high and low perceived control levels. On day 3 of ICU stay, patients who had high levels of perceived control had significantly lower chest pain, fatigue, and dyspnoea compared to those with low perceived control levels. This study found that different levels of perceived control could differentiate the daily symptom experiences of ACS patients. Previous studies addressed this association with different cardiac diagnoses, including heart failure (Lee et al. 2020), and different variables, such as anxiety (AbuRuz 2018), length of stay (AbuRuz and Al‐Dweik 2022), and QoL (Heo et al. 2015). However, this finding was in line with the conclusion of (Lin et al. 2020): that lower levels of perceived control in patients with heart failure are independently correlated with worse symptom status. Based on this, it is highly recommended that CCU clinicians consider interventions that target the enhancement of perceived control (Evangelista et al. 2014).

Patients' perceptions of their clinical symptoms reflect functional abnormalities per se but are also shaped by their experiences of different life situations, cultural backgrounds, and personal self‐efficacy and control. Thus, symptoms are experienced in unique ways even among people with the same symptoms, and these experiences are influenced by a variety of factors, such as age, emotions, and perceived control (Lin et al. 2020; Liljeroos et al. 2018). Previous studies found perceived control to be associated with depressive symptoms and QoL (AbuRuz 2018; Moser and Dracup 1995; Heo et al. 2015), but few studies have been conducted to study symptom status among patients following cardiac events, especially in a prospective manner (Lee et al. 2020; Lin et al. 2020). This highlights the importance of conducting the current study, focused on the most common symptoms experienced by ACS patients: chest pain, fatigue and dyspnoea (Kondo, Oki, et al. 2021; DeVon et al. 2020).

Our findings provide preliminary evidence that ACS patients who had high perceived control levels experienced a significant reduction in the measured symptoms over days of CCU stay. Also, the findings provide independent information about the role of perceived control (i.e., self‐assets) in controlling symptoms among patients, as opposed to the commonly used therapeutic measurements. However, there were no significant differences between the high and low perceived control groups at days 1 and 2 regarding chest pain level, or at day 1 regarding fatigue. This is intuitive, as chest pain and subsequent fatigue are usually at their peak because of ACS accompanying pathophysiological mechanisms (Bergmark et al. 2022). To our knowledge, these specific symptoms were not linked with perceived control levels in prior studies.

While caring for ACS patients, CCU nurses and other clinicians should allocate some additional resources to perceived control. As per our findings, symptom severity is associated with perceived control; given that symptom severity could predict future significant clinical events (Lee et al. 2020), detecting and addressing low perceived control levels might help in reducing the occurrence (and recurrence) of cardiac events. Future RCTs checking the effect of interventions that increase the levels of perceived control on the outcomes of symptom severity among this population are still needed.

### 5.1 Strengths and Limitations

To our knowledge, this is the first prospective study on the relationship between perceived control and symptom experiences in ACS patients to be conducted in an Arab country. Future longitudinal studies may support or modify the effect of perceived control ability in reducing either the recurrence rate or future ACS complications. Despite these strengths, certain limitations should be noted, particularly that symptoms were measured over only 3 days while the cohort study design needs more days to measure the study's outcomes; and the sample size was relatively small, which limits the generalisability of our findings. Also, symptoms severity was measured using a symptoms' diary. However, up to our knowledge, no valid Arabic symptom instrument is available to assess the studied symptoms in ACS patients. Finally, causal associations between perceived control and outcomes cannot be proven, since this is an observational study.

### 5.2 Implications for Practice and/or Further Research

Individual self‐management is a vital part of day‐to‐day care to optimise compliance with treatment and decrease the number of complications. Examining the association between self‐assets (e.g., perceived control levels) and their experienced symptoms could shed light on optimising one's perceived control and other resources. A longitudinal study should be considered when planning future research to examine the effect of such assets on the individual's choice of treatment, adherence to medical plans that could improve health outcomes. Additionally, larger samples of the sub‐groups (STEMI, NSTEMI, and unstable angina) for mixed ANOVA analysis for each sub‐group are highly recommended, since these groups differ in the levels of perceived control, pain, fatigue, and shortness of breath.

## 6 Conclusions

The findings of our study have significant implications in relation to the role of perceived control in reducing symptom severity. We found that patients' day‐to‐day symptom experiences were different in relation to perceived control levels. Compared to those with low perceived control levels, patients who had high levels of perceived control had significantly lower: chest pain on day 3; and fatigue on Days 2 and 3 and dyspnoea on all days. These results indicate that mean levels of perceived control can differentiate ACS patients' daily symptom experiences. Enhancing perceived control levels in ACS patients' symptoms during the course of illness should be the focus of additional clinical research and may serve as the target of future interventions.

## 7 Implications to Advance Nursing Practice

1. Nurses can implement educational interventions that enhance patients' perceived control over their condition, including self‐management strategies, which empower them to engage more actively in their care.
2. Incorporating perceived control into nursing assessments can help identify patients who may benefit from additional psychosocial support.
3. Understanding individual patients' perceived levels of control allows nurses to tailor care plans that incorporate interventions aimed at improving perceived control, such as encouraging decision‐making and involving patients in their treatment plans.
4. By recognising the link between perceived control and symptom severity, nursing care can become more holistic, addressing both physical and psychological aspects of recovery, ultimately leading to better symptom management and patient outcomes.

## Author Contributions

**Fatma Refaat Ahmed:** conceptualisation, methodology, software, validation, formal analysis, investigation, resources, data curation, writing – original draft, writing – review and editing, visualisation, project administration. **Fiona Timmins:** conceptualisation, methodology, software, validation, formal analysis, investigation, resources, data curation, writing – original draft, writing – review and editing, visualisation, project administration. **Rawia Gamil:** conceptualisation, methodology, software, validation, formal analysis, investigation, resources, data curation, writing – original draft, writing – review and editing, visualisation, project administration. **Nabeel Al‐Yateem:** conceptualisation, methodology, software, validation, formal analysis, investigation, resources, data curation, writing – original draft, writing – review and editing, visualisation, project administration. **Mary Ryder:** conceptualisation, methodology, software, validation, formal analysis, investigation, resources, data curation, writing – original draft, writing – review and editing, visualisation, project administration. **Heba Mustafa:** conceptualisation, methodology, software, validation, formal analysis, investigation, resources, data curation, writing – original draft, writing – review and editing, visualisation, project administration. **Mohannad Eid AbuRuz:** conceptualisation, methodology, software, validation, formal analysis, investigation, resources, data curation, writing – original draft, writing – review and editing, visualisation, project administration.

## Ethics Statement

This study has been conducted according to the Declaration of Helsinki 1964. The study was approved by the Research Ethics Committee of the Faculty of Nursing, Alexandria University (approval number: 2022‐9‐34).

## Consent

Each participant signed informed consent before participating in this study.

## Conflicts of Interest

The authors declare no conflicts of interest.

## Data Availability

All data generated or analysed during this study are available from the corresponding author [Fatma Refaat Ahmed] upon request.
